# Cardiovascular events, diabetes and guidelines: the virtue of simplicity

**DOI:** 10.1186/s12933-019-0844-y

**Published:** 2019-03-28

**Authors:** Ricardo J. Esper, Roberto A. Nordaby

**Affiliations:** 1 Buenos Aires University, Buenos Aires, Argentina; 2 University del Salvador, Virrey Loreto 2111, C1426DXM Buenos Aires, Argentina; 3 ACC & AHA, Houston, USA

**Keywords:** Atherosclerosis, Diabetes, Cardiovascular events, Statins, Endothelial dysfunction

## Abstract

Cardiovascular (CV) events or their minor syndromes, as various forms of ischemia, are medical emergencies that do not allow enough time for a guiding anamnesis or proper clinical examination, and lead to relying on Treatment Guidelines, but in many situations it is appropriate to deviate from them. Pathological studies have associated 75% of coronary artery events with atherosclerotic plaque rupture; it is now known that rupture alone is not enough for obstruction or occlusion of the vessel lumen. Concomitant conditions are required for the clinical manifestation of cardiovascular disease, including prothrombogenic and dysfunctional endothelium, less fibrinolytic capacity to protect it, increased platelet activation, increased adrenergic tone, microcirculation vasoconstriction, and other countless factors that contribute to thrombus formation, causing ischemia or infarction. But in most cases, repair of plaque rupture and re endothelization of the lesion are asymptomatic and silent. Atherosclerotic process is a chronic and progressive immune inflammation. Most of the therapeutic indications include statins, which cause side effects in 10% of patients, with a range varying between 7 and 21%, according to different authors. Many investigators have proved that statin use contribute to the genesis of diabetes, reports vary between 1 and 46%, where marked elevation of blood glucose fasting levels and glycosylated hemoglobin have been observed, be it by increased tissue resistance to insulin or by reduced β-cell insulin secretion. Physicians should base their indications on the recommendations provided by Guidelines, but they should not forget that every patient is different, and they should not get confused due to lack of time in an emergency nor be influenced by the latest publications or techniques until they have been properly tested.

*“If it were not for the great variability among individuals, medicine might as well be a science and not an art”*

Sir William Osler

Cardiovascular (CV) events or their minor syndromes, as various forms of ischemia, are medical emergencies that do not allow enough time for a guiding anamnesis or proper clinical examination, and lead to relying on supporting complementary tests, the magic power of recent publications, or the introduction of new techniques, all factors that govern physician behavior, particularly when experience is scarce and generally not based on “real life”. Under such circumstances, relying on Treatment Guidelines is the most common and less risky approach.

Preambles in the Guidelines for Diagnosis and Treatment from scientific organizations always indicate that their main purpose is to satisfy the needs of the majority of patients in all cases. But they also point out that it is the physician who should take the final decision regarding patient care depending on the coexisting circumstances in each patient and the concepts of clinical judgment, or the so-called ‘art of medicine’. On more than one occasion, these two stances (or concepts) may not be identical and may lead to situations in which *“it is appropriate to deviate from the guidelines”*.

Two-thirds of CV events are caused by rupture of atherosclerotic plaques. The remaining third is attributed to endothelial erosion, calcified nodules, and other minor causes [1]. Most CV events are the result of atherosclerosis, a chronic inflammatory disease that begins at a very early age—even in the intrauterine stage—and progresses asymptomatically until the fourth or fifth decade (middle age), when it manifests abruptly as a myocardial or encephalic event or an event in other vascular territories [2].

Although pathological studies have associated 75% of coronary artery events with atherosclerotic plaque rupture, it is now known that rupture alone is not enough for obstruction or occlusion of the vessel lumen. Concomitant conditions are required for the clinical manifestation of CV disease. These conditions include a prothrombogenic, dysfunctional endothelium with little or no fibrinolytic capacity to protect it, increased platelet activation, increased adrenergic tone, arterial vasoconstriction –particularly in the microcirculation–, and other countless factors that contribute to thrombus formation, causing ischemia or infarction. When these conditions do not coexist, the thrombus is most likely lysed normalizing blood flow, the plaque heals and ends up in a fibrous nodule that usually calcifies over time increasing the calcium score in the computed tomography, or may obstructs the vessel lumen to some degree and eventually even rupture generating new inflammation and causing another CV event [3]. But in most cases, repair of plaque rupture and re endothelization of the lesion are asymptomatic and silent [4].

## The inflammatory factor

Atherosclerotic process is a chronic and progressive immune inflammation, but when a CV event occurs, other types of inflammations caused by other mechanisms occur [5].

- The atheromatous plaque caused by the chronic immune inflammation requires acute exacerbation of the inflammatory state with increased activity of metalloproteinase’s for rupture or ulceration, these lesions generate further inflammation intended for repair, necessary but that can become provocative; thus, “inflammation causes more inflammation”.
- Ischemia or necrosis of the affected territory is another source of inflammation to repair such a lesion, but it also amplifies or enhances the process.
- Occasional rescue angioplasty and/or stent implantation are further inflammatory stimuli that progress differently.

All these processes involve inflammatory cytokines triggered by innate immunity cells: monocytes and T lymphocytes, among others. Monocytes are of different types; they are involved in all the inflammatory processes, can migrate from the blood to tissues in response to signals and can differentiate into macrophages, dendritic cells, and foam cells, functioning as process activators or modulators, all key stages in atherogenesis. The same happens with T lymphocytes [6].

Anti-inflammatory therapies acting specifically on macrophages and inflammatory cytokines have been analyzed with monoclonal antibodies [7]. Inhibition of interleukins (IL)-1B showed limited activity in the management of rheumatoid arthritis, but was effective in gout [8]. Tumor Necrosis Factor-α (TNF-α) or IL-6 inhibition evidenced greater impact on rheumatoid arthritis [9]. IL-17 inhibition was effective in psoriasis, but, paradoxically, low levels were associated with increased coronary risk [10].

Over the past decades, dozens of different genes associated with increased coronary disease have been detected, which could translate into new therapeutic goals, including some that affect the metabolism of the LDL cholesterol particles, especially apolipoprotein(a), component of Lp(a) [11]. In addition, the influence of mRNA on epigenetic modifications suggests that these types of molecules could be responsible for residual risk in an ischemic cardiomyopathy despite preventive treatment, and it would similarly explain why patients with risk factors, do not suffer CV events [12].

Continuous communications indicated new factors that influence interaction between inflammation and diabetes: the understanding the relations on nature and nurture [13], promoting health habits from children [14], the newest knowledge of the interaction between endothelial nitric oxide synthetase cholesteryl ester-transfer proteins and angiopoietin-like protein [15], gene expression in peripheral mononuclear cells [16], genetic variant and the altered triglycerides levels [17], and many others that opens new horizons in investigations.

## Therapeutic indications

All the factors described, genetic susceptibility, antigenic substances, endothelium, inflammation, leukocytes, macrophages, different inflammatory cytokines, adrenergic state, vasoconstriction in micro- and macro circulation, balance between thrombogenesis and fibrinolysis, platelet aggregation, in addition to mood, psychic depression, anxiety, fear, and many other not clearly known factors, have to occur in a timely manner to cause a CV event, and it is not always possible to know which of these factors is mainly responsible or, at least, triggers the event [18]. And maybe these factors could also explain that while human beings of all races have atheromatous plaques, only between 0.3 and 1.1% of the general world population suffers a myocardial infarction [19], nearly as many as the sum of traffic accidents [20] and criminal homicides [21].

It is very difficult to guess and even more difficult to identify the determinant factor that causes each CV event, and to be able to select the appropriate therapy to avoid or neutralize it. Therefore, it is uncertain to assume which antiplatelet or anti-inflammatory drug is suitable, or the stimulator of modulating macrophages or inhibitor of inflammatory macrophages, or the monoclonal antibody for the specific cytokines or for blocking the action of the involved metalloproteinase, or even emotional influence as in Takotsubo’s syndrome, which can occur either with feelings of happiness or sadness [22].

To counteract the deleterious effects of CV events, physicians prefer therapeutic interventions that have been tested in large trials, because of the emergency, time is scarce and it is often impossible to find out more about the patient and his particular situation, reperfusion is attempted with mechanical (angioplasty, stenting, etc.) or pharmacological (fibrinolytics, antiplatelet drugs, antithrombotic agents, etc.) techniques. In addition, ACE inhibitors or ARB II agents, beta-blockers, and gastric proton pump inhibitors to resist polypharmacy are administered, together with high statin doses even if the patient is already taking them. Discussions focus on which procedure is most suitable for each form of particular CV event, and the arguments are based on multicenter clinical trials and adherence to Guidelines. In these situations, an important factor is not to rely on statistics of clinical trials but on statistics and experience of the medical center where the treatment procedure will be performed.

On many occasions, due to the generalization of the procedures or the application of Guideline recommendations without taking into account each case in particular, the resulting effect is capricious, multifaceted outcomes not always in line with the results from publications, and patients are often discharged with an indication of multiple-drug therapy without justification.

A frequent long-term consequence is that polypharmacy is maintained without balancing the pros and cons, or because physicians do not have enough time to learn more about the patient or do not want to take risks with the discontinuation of any of the drugs recommended in the Guidelines, even though the recommendation is general or weak.

## Biomarkers and preventive diagnostic techniques

In order to predict CV events, hundreds of molecules involved in this condition have been studied, as they may act as markers to predict the risk for a CV event, and their association has generated encouraging scores with the mathematical results they project. Thus, right from the start, initially with ESR and WBC, later as knowledge progressed included multiple more specific molecules that were discovered as investigations advanced, neopterine, adhesive molecules, interleukins, faze reactants such as fibrinogen and CRP and many others referred to by their acronyms, for example SAA, MPO, PAPP-A, LpPLA2, CPK, CD40L, and many more to which were later added progenitor cells and telomeres length [18], have all been analyzed, but in every case, the increased sensitivity and specificity to detect immediate risk has been low and has not always fulfilled the expectations [23–25]. It is evident that there are so many “predictive markers and/or risk criteria” because none of them is ideal. And who will suffer a CV event, and when, cannot be predicted yet, although it is presumed in those considered higher risk factors.

It is quite evident that the main characteristic of atherosclerosis is the presence of lesions; the predictive value of visualizing those lesions with imaging techniques has been analyzed. All imaging techniques have been tested. The predictive value of visualization of these lesions has been tested. All imaging techniques have been tried [26]. Many of them are non-invasive: ultrasound, vascular echography, flow modulated vascular reactivity and endothelial function, computed tomography, RMN, positron emission, calcium scores, and even very sophisticated tests such as fluorodeoxyglucose and fluorescence of cysteine protease uptake. Others imaging techniques are invasive for example coronary angiography, IVUS, optical coherence tomography and many more. Again, the increase in sensitivity and specificity has not been satisfactory, only slightly better considering the combination of some of them for instance calcium score or RMN and carotid plaque detection [27]. Lately cardiac RMN has been used to detect coronary lesion that could be treated without taking into account confounding statistical factors such as genetic susceptibility or psychological factors [28]. Also, the difference between personalities implies different behaviors, as in the case of depressed and optimistic individuals [29]. Or between those who have achieved expected success and those who have lost a beloved one or suffered a love or work failure [30]. Recently, Cediel et al., showed that stanniocalcin-2 and IGFBP-4 emerge as independent predictors in heart failure patients [31]. In addition, culprit lesions in diabetic patients, may be predicted employing optical coherence tomography [32, 33].

One of the best alternatives to risk prediction is clinical evidence, which can be easily achieved with two questions, two measurements, and two exams: (1) ask the patient if he/she smokes and/or is sedentary; (2) measure waist and blood pressure; and (3) study lipid and glycemic profiles [34]. However, this practice requires time to talk with the patient, and examine and evaluate him/her as a whole. *The virtue of simplicity*.

## The epics of the coronary care unit and medical emergencies

Anyone who is admitted in the Coronary Care Unit due to either a coronary, encephalic or peripheral CV event is treated according to the Guidelines and administered large doses of statins. But in other hospitalizations, such as those due to paroxysmal tachycardia, atrial fibrillation, or simply vasogenic syncope in young people, full doses of statins are also added to the adequate treatment although the Guidelines do not indicate or recommend them. Tolerability and efficacy of statins in the treatment and reduction of CV events both as primary or secondary prevention have been well demonstrated. Statin benefits are mediated by the reduction of LDL-cholesterol fraction levels. Many authors have proposed other benefits, called “pleiotropic”, that are independent from their effect on lipids, although no strong evidence has been provided yet. The studies on these drugs report outstanding effects on prevention, but always in relative values and not always taking into account confounding statistical factors. In absolute values, the event reduction in primary prevention is less than 1%, and little more than 3% in secondary prevention [35].

The difference between primary and secondary prevention is not always accurate because a suspicious precordial pain, ST segment elevation, vascular ultrasound, or non-invasive coronary angiography is enough to move a patient from primary to secondary prevention. Therefore, how can the difference between 1 and 3% be explained? Simply, because those who suffer a CV event probably have other influencing factors, genetic, lifestyle, personality factors, etc. that are still unknown, but make them more vulnerable to CV events.

In a study of the American Heart Association “Get with the Guidelines” Group, Sachdeva et al. showed that 136,905 patients with myocardial infarctions hospitalized in 541 University hospitals in the United States from 2000 to 2006 had total cholesterol levels and their fractions within normal limits for the Guidelines at that time in 75% of cases, 18% had LDL < 70 mg/dl, 10% had HDL > 60 mg/dl, and 21% were under statin therapy [36].

Khot et al. studied the history of cigarette smoking, diabetes, hyperlipidemia, and hypertension, all known risk factors, in 122,458 patients hospitalized due to myocardial infarction. Among them, 19.4% lacked any of the four conventional risk factors, 43% presented only one risk factor, and less than 1% presented the four risk factors [37]. These findings suggest the so-called Helmut Schmidt syndrome or the Winston Churchill syndrome [38]. That does not imply disregarding the risk factors or not treating them properly, but suggests adapting the preventive strategies to make them more convenient according to what each patient needs.

## Evolution of knowledge

Not only do researchers value the qualities of their investigations with great affection, but they also exacerbate their virtues. In the 1980s, dyslipidemia was treated only with fibrates and nicotinic acid, which increase the HDL fraction and are less effective on LDL. All the publications of that time highlighted the importance of this fact and considered LDL less important provided HDL was high [31]. In the early 1990s, the equation was reversed with the advent of statins and the chance to lower LDL. Of course, greater efficacy of statins and learning more about the pathophysiology of atherogenesis played an important role in this process, but the HDL particles were ignored to the point of not being considered in the 2013 ACC/AHA Guidelines, which even led to complaints by European cardiology organizations, that quieten over time [39]. Recent studies show that patients with high HDL have less CV events. Other studies show a U curve, considering that patients are at lower risk with a low or high HDL concentration levels than with acceptable concentration levels, supporting the concept that HDL quality is more important than HDL quantity.

Recently, new proprotein convertase subtilisin/kexin type 9 inhibitors (PCSK9) have appeared that inhibiting the degradation of the LDL cholesterol fraction receptor, allowing a larger amount of receptors to produce greater LDL uptake and > 50% reduction of plasma concentrations compared to baseline values [40]. Since then, multiple criticisms on statin resistance and adverse secondary events have appeared. Is it a coincidence? It is for the reader to judge.

Statins cause side effects in 10% of patients, with a range varying between 7 and 21%, according to different authors [41]. The newer statins have more side effects than the older ones [42, 43]. The most common adverse events include myalgia, myositis with increased creatine phosphokinase (CPK), and rhabdomyolysis in very few cases. Myalgias are not considered significant as long as they do not increase CPK or cause myositis or rabdomyolysis [44]. If the striated muscle hurts it is because it is affected, but how it is affected is still unknown. On the other hand, the myocardium is also a striated muscle but without sensory roots to perceive pain; we do not know to what extent it is not affected as the striated muscle is, and statin therapy has not improved heart failure significantly. Furthermore, the occurrence of dilated cardiomyopathy caused by statins has been reported [45]. In addition, statins do not significantly benefit heart failure treatment [46].

## Coronary arteries and diabetic patients

Statins inhibit the 3-hydroxy-3-methylglutaryl-coenzime-A (HMG-CoA), an intracellular enzyme, which plays a preponderant role in the synthesis of cholesterol. In this way the LDL fraction of cholesterol is effectively decreased and vascular events are reduced, including in diabetic patients. Many investigators have proved that statin use contribute to the genesis of diabetes, reports vary between 1% and 46%, where marked elevation of blood glucose fasting levels and glycosylated hemoglobin have been observed, be it by increased tissue resistance to insulin or by reduced β-cell insulin secretion [47, 48].

This effect has been observed in nearly all the large studies, with more or fewer consequences according to which statin was used. Different mechanisms have been mentioned as the possible cause of this effect, reduction of protein geranylgeranylation, diminished phosphorylation of AKT insulin receptor substrate, 3β glycogen synthetase kinase, a reduced GLUT4 regulation, all these factors reduce tissue glucose phosphorylation uptake [49].

Other studies have shown that statins increase Interleukin -Iβ, which influences the inflammasome activation, a mechanism that increases the adipocyte and adipose tissues’ resistance to insulin. Insulin production by the β-cells of the pancreas requires calcium influx through calcium sensitive channels controlled by ATP activated by potassium channels. In this way, through mechanisms involved in inflammation they contribute to insulin resistance and/or a reduction in insulin secretion [50–52].

Cerderberg et al. in a 6-year follow-up study of patients treated with different doses of all the statins currently prescribed, observed 46% increased risk of diabetes due to enhanced resistance and/or reduced insulin production [53]. If we consider that between one quarter and one-third of the adult population in the United States takes statins, such a marked increase of diabetes over the past years might be due, in part, to statins, or at least be a confounding statistical factor.

Both statins and PSCK9 inhibitors increase insulin resistance or reduce insulin release, which could increase the risk of diabetes in predisposed individuals. Two recent publications on PCSK9 genetic variants with mendelian randomization and meta-analysis reported increased glucose, increased weight, and increased diabetes with this medication [54]. Ravnskov et al. evaluated 68,000 patients from 30 cohorts and found no benefit of statin therapy in primary prevention to people over 60 years of age, and in secondary prevention for people over 75 years of age [55]. Other studies report similar results with different cohorts [56].

The problem of the increase in diabetes appears to be not only due to statin side effects, but are apparently shared with the new PCSK9. It has been suggested that cholesterol and glucose have several points in common that can influence each other. Perhaps in future studies more light can be thrown on this subject [43, 54].

All human cells membranes have between 0.5 and 0.9% cholesterol, except the crystalline lens were the concentration is 3%, marked reduction in cholesterol concentration has not been investigated as a cause of cataracts [55].

However, none of these observations reduce the effectiveness and usefulness of statins in primary and secondary prevention of CV events. It is only important to remember them so as not prescribing them to patients who are not at risk, not only when they are discharged from the Coronary Care Unit but also in a simple medical consultation, because it leads to loss of confidence in a drug that, if properly administered, is very useful [56–59].

It seems that statins have “a Yin and a Yang” made up of their benefits and their adverse effects; evidence shows that they have more benefits than drawbacks. It is not a question of putting statins in the running water of cities but of prescribing them to the adequate populations. We have to counteract the physician’s inertia.

## Conclusion

The best way to prevent a CV event will be achieved by educating the population from childhood, teaching people how to eat healthily, avoid overweight and physical inactivity, and to eradicate smoking. Scientific Cardiology Societies have committed to a 25% reduction of CV events in 25 years, and it will not be achieved with medication but with a healthy lifestyle. Physicians should base their indications on the recommendations provided by Guidelines, but they should not forget that every patient is different, and they should not get confused due to lack of time in an emergency nor be influenced by the latest publications or techniques until they have been properly tested [60, 61].

Always remember Norman Cousin’s teachings: “Diagnosis belongs to science; Prognosis belongs to patients.”

## Abbreviations

CV: cardiovascular events
IL: interleukins
TNF: Tumor Necrosis Factor
Lp_a_: Lipoprotein L_a_
ACE: Angiotensin Converting Enzime
ARB: Angiotensin Receptor Blocking
ERS: Eritrocytes Sedimentation
WBC: white blood cells
CRP: C reactive protein
RMN: Resonance Magnetic Nuclear
IVUS: Intravascular Ultrasound
LDL: Low Density Lipoprotein
HDL: High Density Lipoprotein
PCSK9: proprotein convertase subtilisin/kexin type 9 inhibitors
CPK: creatine phosphokinase
HMG-CoA: 3-hydroxy-3-methylglutaryl-coenzime-A reductase
